# Spatial distribution of uptake of newly introduced vaccines and its associated factors among children aged 12–35 months in Ethiopia: Multi-scale Geographically Weighted Regression Analysis

**DOI:** 10.1371/journal.pone.0345899

**Published:** 2026-07-02

**Authors:** Habtamu Guaguahu Feleke, Mulat Belay Simegn, Daniel Tarekegn Worede, Werkneh Melkie Tilahun

**Affiliations:** 1 Department of Public Health, College of Medicine and Health Sciences, Debre Markos University, Debre Markos, Ethiopia; 2 Department of Theory and Empiricism of Healthcare, Institute of Social Work University of Kassel, Kassel, Germany; Haramaya University College of Health and Medical Sciences, ETHIOPIA

## Abstract

**Background:**

The pneumococcal conjugate vaccine (PCV) and rotavirus vaccine (RVV) have been introduced to Ethiopia’s expanded childhood immunization program in 2011 and 2013, respectively.

**Methods:**

A cross-sectional study among 2,055 children aged 12–35 months was employed. Data were extracted from the Kids Record file of the 2019 Ethiopian Mini Demographic and Health Survey. Spatial regression models were fitted and compared using corrected Akaike Information Criteria, Bayesian Information Criteria and adjusted R^2^. Spatial predictors were determined to be statistically significant if their p-value was < 0.05.

**Results:**

Incomplete uptake of recently introduced immunizations was observed in nearly half (48.83%) of children aged 12–35 months. Its distribution throughout Ethiopia’s regions shows significant spatial clustering, with the eastern part of SNNPR and Somali regions having hot spots. A total of 3 significant clusters, located in southern Oromia, east and west Hararge, the entire SNNPR, and the majority of the Somali region, with a high rate of incomplete uptake of newly introduced vaccines, were identified during SaTScan analysis. Not having vaccination cards, household size more than 5 members, home delivery, not having postnatal care, and less than 18 maternal age at first birth are positive significant spatial factors while parents not being head of the household were identified as negative significant spatial factors.

**Conclusion:**

There is high incomplete uptake and spatial disparities of PCV and RVV. To improve vaccination coverage among children aged 12–35 months, policymakers and health planners should prioritize targeted interventions in hotspot areas, strengthen maternal health service utilization, and enhance vaccination tracking systems. Promoting community awareness and improving access to essential health services will be critical to ensuring equitable immunization uptake across the country.

## Introduction

Immunization is a primary intervention for preventing and controlling infectious diseases, which declines the prevalence of immune-preventable diseases [1–4]. Every year, nearly 2 million children under five die globally from pneumonia and diarrhea [5,6]. In developing nations, diarrhea leading cause of under-five fatalities, with rotavirus being the primary driver of severe diarrheal disease [7–9]. In Ethiopia, pneumonia and diarrhea are leading causes of illness and death in young children [10–12]. Rotavirus accounts for about 28% diarrheal hospitalizations and 6% diarrheal deaths among under-fives [13–17]. The high economic cost of managing these vaccine-preventable diseases underscores the need for prevention [1,18–20]. PCV and RVV vaccines effectively reduce the burden of diarrheal gastroenteritis and pneumonia in Ethiopian children [21,22].

Previous studies revealed that disparities in wealth status [3,23–29], birth order [19,26,29], sex of the child [25,30], maternal level of education [3,23,24,26–29,31–33], number of antenatal care (ANC) visits [3,25,26,28,29,31], marital status [3,24,30,31,34], place of delivery [3,24–26,28,29,31–33,35], mode of delivery [25,35], postnatal care (PNC) within two weeks of delivery [3,27–29,36,37], availability of a vaccination card at home or health facility [30,31,33,37–43], maternal age [29,30,44–47], place of residence [24,31,32,48,49], number of under-five children [3,50–52], relationship with household head [53], sex of household head [19], parity [52], maternal age at first delivery [54,55], pregnancy status [49], twin delivery [56], number of family members [25,49,52,57–59], healthcare access, household factors, computing health priorities, and cultural beliefs contribute to the uneven distribution of vaccine uptake [60–66].

Past measures taken to overcome the problem include efforts to strengthen the immunization supply chain, increase access to vaccination services through mobile clinics and community outreach programs, improve public awareness campaigns about the value of vaccination, and involve influential people and community leaders in encouraging immunization uptake [67–69]. Despite these interventions, pneumonia and diarrheal diseases continue to be the primary causes of morbidity and mortality among children in Ethiopia, highlighting the need for more targeted (area-specific) and long-run strategies [70–72]. Such strategies can be reached after understanding the spatial patterns (hotspots and spatially varying significant covariates) of vaccine uptake and identifying outreach communities.

Policymakers and public health professionals may develop specific strategies and address the distinct issues that each region faces, especially for underserved and hard-to-reach populations, by identifying the spatial pattern and contributing factors. This will also guarantee more equitable use of PCV and RVV vaccinations. Thus, the current study aimed to investigate the spatial distribution of newly introduced vaccine uptake and its spatial determinants among children aged 12–35 months in Ethiopia.

## Method and materials

### Data sources, setting, population, and sampling design

The 2019 Ethiopian Mini Demographic Health Survey (EMDHS) is a nationally representative household survey conducted from March 21 to June 28, 2019, across nine regions and two city administrations. A two-stage stratified sampling technique was employed. Each region was stratified into urban and rural areas, yielding 21 sampling strata. In the first stage, a total of 305 enumeration areas (EAs) (93 urban and 212 rural) were selected independently within each stratum using probability proportional to EA size, based on the 2019 Ethiopian Population and Housing Census frame. Sample allocation was designed such that a similar number of EAs (25 EAs) were selected from eight regions, regardless of population size, to ensure comparable precision, while 35 EAs were selected from the three larger regions (Amhara, Oromia, and Southern Nations, Nationalities, and Peoples Region [SNNPR]).

In the second stage, a fixed number of 30 households per cluster were selected using systematic random sampling from updated household listings. All women aged 15–49 years who were either permanent residents or visitors who stayed in the selected households the night before the survey were eligible for interview. Anthropometric measurements were also collected from children aged 0–59 months. For this study, data were extracted from the Kids Record (KR) file, and a weighted sample of 2,055 children aged 12–35 months was included in the analysis. This age group was considered because the EMDHS Kids Record data set provides vaccination information only for children younger than 36 months.

### Variables of the Study

#### Outcome variable.

The primary outcome variable of this study was the uptake of newly introduced vaccines among children aged 12–35 months. Specifically, we assessed the proportion of children who had not received the complete recommended doses of the Pneumococcal Conjugate Vaccine (three doses at 6, 10, and 14 weeks) and the Rotavirus Vaccine (two doses at 6 and 10 weeks of age) [63,72–74].

#### Independent variables.

The explanatory variables included in the study were selected based on a literature review and professional recommendations. These variables were wealth index, birth order, sex of the child, maternal education, number of ANC visits during pregnancy, marital status, place of delivery, mode of delivery, PNC within two weeks of delivery, availability of a vaccination card in the home or at a health facility, maternal age, place of residence, number of under-five children, relationship with household head, sex of household head, parity, maternal age at first delivery, pregnancy status, twin siblings, household head age, and household Size (**Table 1**).

#### Measurement.

**Table 1:** Measurements of explanatory variables in the Study, 2019.

Variables	Measurements
Wealth status	Proportion of children from poor wealth index
Birth order	Proportion of children with birth order >7
Maternal education	Proportion of children whose mother didn’t attend formal education
ANC visits during pregnancy	Proportion of children whose mothers have inadequate ANC visits (less than 4 visits)
Marital status	Proportion of children whose mother is currently not married or not living with man
Place of delivery	Proportion of children born in a health facility (government hospital, government health centre, government health post, private hospital, private clinic, non-governmental health facility)
Mode of delivery	Proportion of child delivered by cesarean section
PNC visit	The proportion of children with no PNC visit occurred within 2 months of delivery.
Availability of a Vaccination card	Proportion of children without a vaccination card
Sex of the child	Proportion of male children
Maternal Age	Proportion of children with maternal age less than 25 years
Place of residence	Proportion of children who live in rural area
Number of under 5 children	Proportion of children who live with more than one under-5 child in the house
Relationship with household head	Proportion of children whose parent are not the household head
Household Sex	Proportion of children within female headed house
Parity	Proportion of children with 5 and more parity mothers
Maternal age at first delivery	Proportion of children with mothers who are less than 18 years old at the first delivery
Pregnancy status	Proportion of children whose mother is pregnant
Twin siblings	Proportion of children who have twin
Household head age	Proportion of children in a household whose head age is more than 35 years old.
Household Size	Proportion of children who live in a house with more than 5 members.

### Data management and analysis

Socio-demographic characteristics of the study participants are calculated with proportions for categorical variable and mean ± SD, or Median ± IQR for continuous variables, and presented in a table.

### Analyzing pattern and mapping clusters

#### Spatial autocorrelation analysis.

A global Moran’s I was used to determine the spatial distribution of PCV and RVV uptake among Ethiopian children between the ages of 12 and 35 months. Its value ranges from −1–1. Values approaching +1 show that neighbouring places typically have similar values (clustering). Spatial patterns are random when values are near zero. Values approaching −1 indicate that values in the vicinity typically have different values (dispersion).

#### Hot spot analysis.

We performed a local hot spot analysis using Getis-Ord Gi*statistic to complement the Global Moran’s I analysis and investigated the spatial pattern of PCV and RVV uptake at the local level. The Getis-Ord Gi* statistic identifies statistically significant hot spots and cold spots within the country.

#### Spatial interpolation.

We used the spatial interpolation technique to estimate the possible PCV and RVV uptake levels for areas in the country that were not sampled. To do this, we used the Empirical Bayesian Kriging (EBK) interpolation, a geostatistical technique that uses the spatial autocorrelation structure of the sampled EAs [75,76].

#### Spatial scan analysis.

Bernoulli-based model spatial scan analysis using Kuldorff’s SaTScan version 9.6 software was carried out to identify significant clusters of incomplete PCV and RVV vaccination uptake. In a circular scanning window, children who had not taken complete PCV and RVV doses were classified as cases. And those who had complete vaccination for PCV and RVV were classified as controls. An upper bound was set at a maximum spatial cluster size of less than 50% of the population. Most likely clusters that were found using likelihood ratio tests and consistent significance levels at different Monte Carlo replications (0, 9, 999, 9999 and 99999) were presented.

### Spatial regression analysis

To identify relationships between incomplete PCV and RVV uptake among Ethiopian children and explanatory variables, we performed spatial regression analyses. We began by identifying a set of potential explanatory variables that could influence the uptake of PCV and RVV. Then, among candidate combinations of variables, the best combination model was compared by adjusted R2 and Akaike Information Criteria (AIC). The global ordinary least squares model was fitted, then model assumptions were tested, and further advanced models were fitted to control the spatial dependencies in the data.

#### Spatial exploratory regression and Ordinary Least Squares (OLS) regression.

A spatial exploratory regression analysis was done to determine possible combinations of candidate explanatory variables. Then, OLS regression on these variable combinations and model diagnostics was done. Variance Inflation Factor (VIF): to check for multicollinearity among the explanatory variables. To assess the normality of the model residuals, the Jarque-Bera statistic was used, because it is convenient and commonly available in software, despite being conservative (reducing type I error) and having lower power compared to alternatives [77,78]. Joint F and Wald statistics: to evaluate the overall significance of the model. Koenker statistic: check for non-stationarity in the spatial processes. In the end, we checked for spatial autocorrelation in the model residuals.

#### Geographical weighted beta regression, spatial lag, spatial error model and multi-scale geographical weighted regression.

Geographically weighted beta regression (GWBR), Spatial error model (SEM), and spatial lag model (SLM) were considered and compared based on their Bayesian Information Criteria (BIC) and corrected Akaike Information Criteria (AICc) values. The explanatory variables are similar to those included in the OLS. R version 4.4.3 using the “gwbr” package was used to estimate the GWBR model’s parameter at each enumeration area, and for the spatial lag and error model, the “spatialreg” package was used.

In both SEM and SLM, the spatial weights matrix was constructed using the Inverse Distance Weighting (IDW) method. Specifically, geographic coordinates from each spatial unit were extracted from projected spatial data. A full pairwise Euclidean distance matrix was computed, and self-distances were excluded by setting diagonal entries to missing. The resulting inverse distance matrix was used to estimate both SLM and SEM models via maximum likelihood using the “lagsarlm” and “errorsarlm” functions from the spatialreg package in R. The model equation for the spatial lag model and spatial error model is shown in Equation (1) and Equation (2), respectively [79].


$$\begin{array}{c} Y=\;\rho W_{y}+X\beta +\;\varepsilon \end{array}$$ (1)

$$\begin{array}{c} Y=X\beta +\;\varepsilon \end{array}$$ (2)

$$\begin{array}{c} where,\;\varepsilon =\lambda W_{\varepsilon }+u \end{array}$$

Where, in the spatial lag model, *W*_*y*_ is the spatially lagged dependent variable (weighted average of neighbouring values of *Y*) and *ρ* is the spatial autoregressive coefficient, representing the strength of spatial dependence. In the spatial error model, *W*_*ε*_ is the spatially correlated error term, is the spatial lag of the error term, 𝜆 is the spatial error coefficient, capturing the degree of spatial autocorrelation in the errors, and *u* is a vector of independently and identically distributed error terms with zero mean and constant variance.

Because the dependent variable was bounded between 0 and 1, and the GWBR model was fitted. Predictor variables included are those included in OLS. Given that the EAs are clustered, Weights are determined by a method known as the adaptive bi-square kernel distance decay function [76,80]. Optimal bandwidth selection was performed using the Golden Section Search (GSS) algorithm, based on the minimization of the AICc [76,81].

The adjusted R^2^ and AICc values were used to compare the Spatial models and OLS models. On the best model, residual autocorrelation was tested, and then further model advancement to Multi-scale Geographic Weighted Regression was performed. The GWBR was the best-fit model based on the AICc; however, its residual is not normally distributed. So, Multi-scale Geographically Weighted Regression (MGWR) using the “GWmodel” package in R was considered. GWBR assumes that all explanatory variables influence the outcome at the same spatial scale. In contrast, MGWR relaxes this assumption by allowing each variable to operate at its own spatial scale. MGWR does this by estimating a separate, optimal bandwidth for each covariate, indicating the specific spatial range over which that variable has an effect. This makes MGWR more informative and flexible, as it captures the varying spatial influences of different predictors rather than applying a uniform scale to all [82]. The model equation for GWBR and MGWR is shown in Equation (3) and Equation (4), respectively [82].


$$\begin{array}{c} y_{i}\;=\sum_{j=0}^{m}\beta_{j}\left(u_{i},v_{i}\right)x_{ij}+\;\varepsilon_{i} \end{array}$$ (3)

Where *X*_*ij*_ is the *j*^*th*^ predictor variable, *β*_*j*_ *(u*_*i*_*,v*_*i*_*)* is the *j*^*th*^ coefficient, *ε*_*i*_ is the error term, and *y*_*i*_ is the response variable.


$$\begin{array}{c} y_{i}\;=\sum_{j=0}^{m}\beta_{bwj}\left(u_{i},v_{i}\right)x_{ij}+\;\varepsilon_{i} \end{array}$$ (4)

Where *bwj* in *β*_*bwj*_ indicates the bandwidth used for calibration of the *j*^*th*^ conditional relationship.

### Missing values

Missing data when calculating proportions were managed according to the Demographic Health Survey (DHS) guidelines, which are specific to each covariate (either excluding from the numerator or excluding from the numerator and denominator while calculating the proportion) [83].

### Ethical considerations

Since the study was a secondary data analysis based on a publicly accessible DHS database, participant participation, and ethical approval were not required. Nevertheless, we asked the MEASURE DHS Program for the data, and we were given permission to download and utilize it. All analyses were conducted in compliance with the DHS data use guidelines to maintain the privacy and rights of the survey participants.

## Results

Our Study included a total of 2,055 weighted children aged 12–35 months. The level of incomplete PCV and RVV vaccination among these children is 48.83% in Ethiopia. About three-fourths (74.86%) of children’s mothers were aged 25 and above. Half (49.4%) of the children’s mothers had attended at least primary education. Almost all (95.05%) of children’s mothers live together with their partner. About 58.33% of children were living in a house with more than one under-five children. The majority (72.71%) were living in rural areas of Ethiopia, while about 40.58% were residing in the oromia region. About 56.88% of children’s mothers didn’t have adequate ANC visits during pregnancy. However, 87.01% of children have had PNC within 2 months. Half (50.62%) of the children were born at home. The mean age of the mothers at their first delivery is 27.75 ± 6.34 years old (**Table 2**).

**Table 2:** Socio-Demographic characteristics of study participants and their mothers, Ethiopia, 2019.

Variables	Category	Frequency	Percentage
Age of the mother	≥25years	1538	74.84
	<25years	517	25.16
Highest educational level	At least primary education	1040	50.60
	No education	1015	49.40
Current marital status	Didn’t live together	102	4.96
	live together	1953	95.04
Place residence	Urban	561	27.29
	Rural	1494	72.71
Region	Tigray	138	6.72
	Afar	32	1.58
	Amhara	410	19.94
	Oromia	834	40.58
	Somali	130	6.32
	Benishangul	24	1.16
	SNNPR	395	19.23
	Gambella	8	0.39
	Harari	6	0.30
	Addis Ababa	66	3.22
	Dire Dawa	11	0.55
Sex of the household head	Male	1786	86.90
	Female	269	13.10
Wealth index	Moderate and above	1161	56.49
	Poor	894	43.51
Under 5 children	No	856	41.65
	Yes	1199	58.35
Place of Delivery	Health Facility	1015	49.39
	Home	1040	50.61
Number of antenatal visits(ANC) during pregnancy	Adequate(≥4 visit)	774	43.11
	Inadequate(<4 visit)	1021	56.89
PNC	Yes	1788	87.00
	No	267	13.00
Age of respondent at 1st birth	18 & After	1242	60.43
	Before 18	813	39.57
Currently pregnant	No	1795	87.34
	Yes	260	12.66
Parents are not the head of the household	Yes	168	8.2
	No	1887	91.8
Availability of vaccination card	Yes	593	28.85
	No	1462	71.15
Household members size	5 & below	1005	48.90
	Above 5	1050	51.10

### Spatial distribution of PCV and RVV uptake

Moran’s Index value of 0.39 with a Z-value of 8.39 and a P-value of less than 0.001 confirms that the existing spatial pattern is statistically significant clustering. In particular, there are certain Enumeration areas with similar incomplete PCV and RVV uptake levels (high-high or low-low) that are clustered.

### Hot Spot analysis

**Fig 1:**
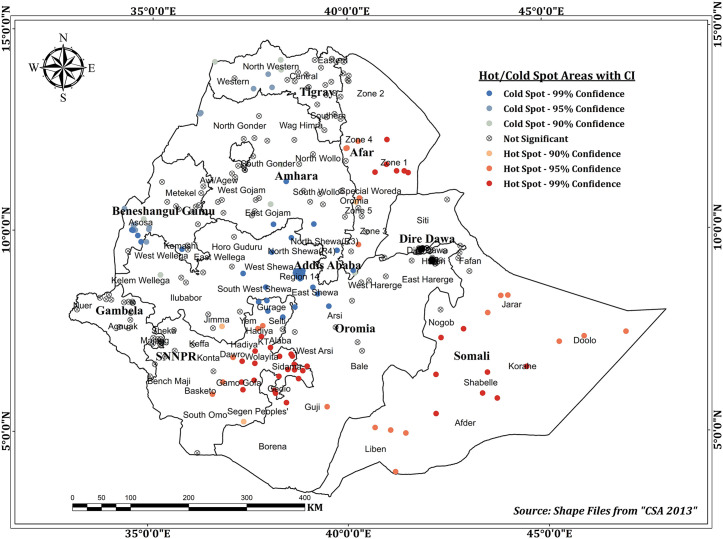
Hot Spot analysis of incomplete newly introduced childhood vaccination among children aged 12–35 months in Ethiopia, 2019. Map image is the intellectual property of Esri and is used herein under license. Copyright © 2026 Esri and its licensors. All rights reserved.

The Hot Spot analysis showed significant cold spot areas in Benishangul, Addis Ababa, and Shewa. Areas like Dire Dawa, Hareri, Gambela, Amhara, and Tigray regions are presented as not significant. Whereas, Afar, Somali, and the North East part of the SNNPR regions showed hot spot areas indicating these geographical areas are with EAs with high incomplete PCV and RVV vaccination uptake surrounded by other EAs of high incomplete uptake (Fig 1).

### Empirical Bayesian Kriging interpolation

**Fig 2:**
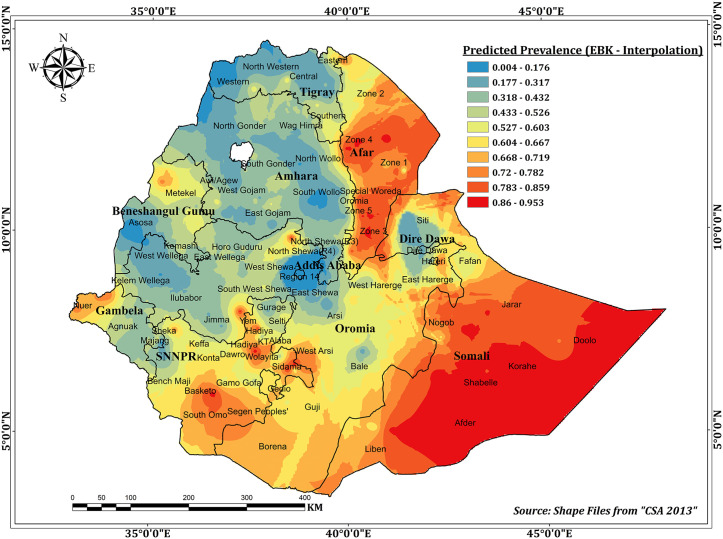
Interpolated prevalence of incomplete newly introduced childhood vaccination among children aged 12–35 months in Ethiopia, 2019. Map image is the intellectual property of Esri and is used herein under license. Copyright © 2026 Esri and its licensors. All rights reserved.

The Somali region stands out as a high-risk area, with the map showing a predominance of dark red shading, indicating the highest predicted prevalence range (72.0% − 95.34%) of incomplete uptake of these vaccines. The Afar region also exhibits a significant portion of high-risk areas, with red (66.8% to 95.34) and in its northern part of ranges somewhat 52.7% − 66.7%. In contrast, the Amhara, Tigray, Benishangul Gumuz, Addis Ababa, Shewa, the eastern part of Gambella, Dire Dawa, and the western parts of the Oromia region show lower predicted prevalence, as indicated by the blue shades marked with less than 43.3% level. Western part of Gambela, north-east part of benshangul gumz (Metekel), the southern and western part of Oromia, and almost all of the SNNPR region display medium-risk areas that range from 52.7% − 66.7% predicted prevalence of Incomplete newly introduced vaccine uptake. Additionally, specific districts in the South Omo and Wolayita zones (Boloso Sore, which is located south of Omo Sheleko) of SNNPR also show a high risk for not having the full dose of newly introduced vaccines (Fig 2).

### Spatial scan analysis

**Fig 3:**
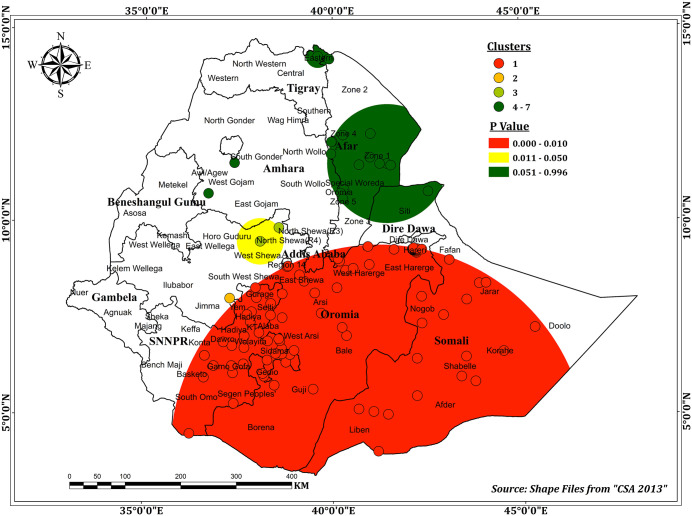
Spatial scan analysis of incomplete newly introduced childhood vaccination among children aged 12–35 months in Ethiopia, 2019. Map image is the intellectual property of Esri and is used herein under license. Copyright © 2026 Esri and its licensors. All rights reserved.

The spatial scan analysis was performed at different Monte Carlo replications (0, 9, 999, 9999 and 99999) and identified consistently three significant clusters. These clusters have a total of 98 enumeration areas; out of those, 95 enumeration areas were included in the primary cluster. Whereas one enumeration area was a secondary cluster solely, and two enumeration areas were in the third cluster. The primary cluster was found in southern oromia, east and west hararge, all SNNPR except the western part, and the entire Somali region (4.028421 N, 41.180723 E) / 601.08 km radius as shown in Fig 3. Children residing in this cluster were 83% more likely to have incomplete doses of newly introduced vaccines (Relative Risk (RR) = 1.83, Log Likelihood Ratio (LLR) = 85.3, P value < 0.001 compared to children outside the window. The secondary clusters were found in the oromia region (8.039877 N, 37.283375 E) / 0 km radius. Children residing in this cluster were 2.08 times more likely to have incomplete doses of newly introduced vaccines (RR = 2.08, LLR = 15.29, P value = 0.001 compared to children outside the window, however, this enumeration area was not a significant hot spot in Getis-Ord Gi* hot spot analysis what if the empirical Bayesian Kriging interpolation resulted in above 66.8% of incomplete PCV and RVV uptake. The third cluster was also found in the oromia region, northern part (9.531226 N, 38.081685 E) / 67.38 km radius. Children residing in this cluster were 67% more likely to have incomplete doses of newly introduced vaccines (RR = 1.67, LLR = 8.58, P value = 0.023 compared to children residing outside the window (Fig 3 and S1 Table).

### Exploratory spatial regression

We have used a total of twenty-one different variables based on their significance in previous literature and professional recommendations. Accordingly, three possible combinations were considered, and one combination was selected as better based on adjusted R^2^ and AICc. All the models, OLS, GWBR, spatial lag, spatial error, and MGWR, included only these mentioned variables. These combinations were the proportion of children whose parents are not the head of the house, live with more than 5 household members, whose mothers had their first delivery at less than 18 years old, did not have a PNC visit in their first two months and the proportion of children who didn’t have a vaccination card (Table 3).

**Table 3:** Exploratory spatial regression variable combinations for incomplete newly introduced childhood vaccination among children aged 12–35 months in Ethiopia, 2019.

Variable Combinations	Adjusted R^2^	AICc
Relationship with household head, Availability of a Vaccination card, PNC visit, Maternal age at first delivery, Household Size, Place of delivery	0.67	−113.82
Number of under 5 children, Place of residence, Relationship with household head, Availability of a Vaccination card, PNC visit, Maternal age at first delivery, Household Size, Place of delivery	0.67	−113.49
Number of under 5 children, Relationship with household head, Availability of a Vaccination card, PNC visit, Maternal age at first delivery, Household Size, Place of delivery	0.67	−113.43

### The ordinary least square regression analysis results

The OLS model was statistically significant, as indicated by the high F-statistic and Joint Wald Statistic, both of which have P values less than 0.05. The model explains a relatively large proportion of the variance in proportion of incomplete PCV and RVV uptake with a Multiple R^2^ value of 0.6820 and an Adjusted R^2^ of 0.6756, suggesting the independent variables account for around 67.56% of the variation in the outcome. However, the diagnostics also reveal some potential issues with the model assumptions. The Koenker Statistic and Jarque-Bera Statistic were significant (P < 0.05), indicating violations of the residuals’ assumption of constant variance and normality, respectively. The Global Moran index I for the OLS residuals was 0.082 with a z-value of 3.34 and p-value of ≤ 0.001, which shows the existence of spatial autocorrelation of residuals. The explanatory variables in the original OLS model had a VIF value of less than 7.5, indicating that multicollinearity was not a significant issue. Overall, the existence of significant residual spatial autocorrelation suggests further spatial model advancement like GWBR, Spatial Lag Model, and Spatial Error model (Table 4).

**Table 4:** The OLS regression analysis results for incomplete newly introduced childhood vaccination among children aged 12–35 months in Ethiopia, 2019.

Variable	Coefficient	Robust_SE	Robust_Pr	VIF
Intercept	−0.000495	0.063724	0.993808	-------
Proportion of children with no head parents	−0.202267	0.064834	0.001999*	1.098563
Proportion of children with a mother aged less than 18 at first delivery	0.131203	0.059578	0.028408*	1.207527
Proportion of children with no PNC	0.140917	0.070350	0.046075*	1.149849
Proportion of children with no vaccination card	0.671191	0.043284	≤ 0.001*	1.657797
Proportion of children with >5 house members	0.117960	0.048977	0.016618*	1.336526
Proportion of children has been delivered at home	0.180288	0.045417	≤ 0.001*	1.886526
**OLS Diagnostics**				
**Diagnostic Parameters**	**Values**		**P-Values**	
**Number of Observations**	303		−	
**Joint F-Statistic**	105.80		0.001*	
**Joint Wald Statistic**	1231.49		0.001*	
**Koenker Statistic**	32.48		0.001*	
**Jarque-Bera Statistic**	19.07		0.001*	

Robust_SE: Robust Standard Error, Robust_Pr: Robust Probability Value, VIF: Variance Inflation Factor, OLS: Ordinary Least Squares, F-Statistic: Fisher’s Statistic. PNC: Postnatal Care.

### Geographically weighted beta regression, spatial lag and spatial error model

The GWBR model showed a substantially improved fit, with the AICc decreasing from −114.31 to −616.39 (difference in AICc = 502.08). However, the Global Moran’s I statistic for the model residuals was 0.114 (p = 0.012), indicating the presence of significant residual spatial autocorrelation. To account for this residual spatial autocorrelation, further spatial modeling was employed using both the SLM and SEM.

In the spatial lag model, there was a statistically significant spatial lag parameter (ρ = 0.006, p < 0.001), indicating the presence of structural spatial dependence in incomplete PCV and RVV vaccinations uptake. The Likelihood Ratio (LR) test for spatial lag dependence yielded a p-value < 0.001, and the model has an AICc value of −128.13, confirming that the lag model is better compared to the global OLS method. The Wald statistic for ρ was also highly significant, supporting the inclusion of the spatial lag term. Additionally, the Lagrange Multiplier (LM) test for residual autocorrelation resulted in a test value of 8.88 (p = 0.003), suggesting that residual spatial dependence remains even after accounting for the lag structure.

In the spatial error model, there was a statistically significant spatial error parameter (λ) with a value of 0.315 with a p-value of 0.003, indicating strong evidence of spatial autocorrelation in the error terms. The LR test for spatial error dependence yielded a p-value of 0.004, affirming the appropriateness of the SEM. The AIC was –124.22, lower than the OLS model, suggesting a better model fit than the OLS model. As shown in (Table 5), model comparison based on AIC indicated that the GWBR model provided the best overall fit among other models (OLS, SLM, and SEM). However, the residuals from the GWBR model still exhibited significant spatial autocorrelation, suggesting that spatial dependence was not fully accounted for. Therefore, to better capture spatial heterogeneity and remaining spatial structure, a multi-scale geographically weighted regression (MGWR) model was subsequently fitted (Table 5).

**Table 5:** Comparison of spatial regression models for incomplete newly introduced childhood vaccination among children aged 12–35 months in Ethiopia, 2019.

Models	AIC	BIC
Ordinary Least Square Model	−114.31	−84.59
Geographically Weighted Beta Regression Model	−616.39	−586.43
Spatial Lag Model	−128.13	−94.71
Spatial Error Model	−124.22	−90.80

### Multi-scale geographical weighted regression

For better accounting of spatial heterogeneity, the MGWR model was employed. The predictor variables considered in this model include; proportion of children whose parents are not the head of the house, live with more than 5 household members, whose mothers had their first delivery at less than 18 years old, did not have a PNC visit in their first two months and the proportion of children who didn’t have a vaccination card. Using the “GWmodel” package in R, the MGWR model was calibrated with a bisquare kernel and an adaptive bandwidth selection to accommodate the uneven spatial distribution of data points. The model was optimized using the AIC criterion. Each predictor was allowed to operate at its own spatial scale, as shown in (Table 6), reflecting the principle that different factors may influence vaccination outcomes over different geographic extents.

**Table 6:** Multi-scale Geographically Weighted Regression (MGWR) model result for predictors of incomplete newly introduced childhood vaccination among children aged 12–35 months in Ethiopia, 2019.

Predictor Variable	Minimum	1st Quartile	Median	3rd Quartile	Maximum	Bandwidth
**Intercept**	−0.133	−0.083	0.011	0.113	0.155	149
**Proportion of early first births (<18)**	−0.065	0.036	0.097	0.186	0.557	85
**Proportion with no postnatal care**	0.017	0.040	0.063	0.090	0.121	279
**Proportion without a vaccination card**	0.509	0.539	0.5825	0.6129	0.648	263
**Proportion of home deliveries**	0.172	0.199	0.207	0.212	0.228	286
**Proportion where the parent is not the household head**	−0.093	−0.101	−0.103	−0.105	−0.109	301
**Proportion of large households (>5 members)**	0.092	0.096	0.097	0.098	0.099	301

The model diagnostics revealed a good fit, with an R-squared of 0.737 and an adjusted R-squared of 0.706, indicating that a substantial proportion of spatial variability in incomplete vaccination coverage was explained by the selected covariates. The residual sum of squares was 9.56, and the AIC was −163.49. A Moran’s I test on the residuals yielded no evidence of significant spatial autocorrelation (Moran’s I = −0.0023, P = 0.476), affirming that the model effectively captured underlying spatial patterns in comparison to all the above models (Table 6).

The MGWR results showed that early maternal age at first delivery had a spatially varying effect on incomplete vaccination uptake (β: −0.065 to 0.547). However, statistically significant positive associations were observed only in specific areas, including parts of Amhara (South Wollo and North Shewa), Afar (Zones 1, 3, and 5), Eastern Hararghe (including Dire Dawa and Harari), and the Somali region (Jijiga: Nogob, Fafan, and Siti zones) (S1 Fig). Lack of postnatal care also demonstrated substantial spatial variation (β = 0.005 to 0.739). Statistically significant association is seen specifically in the Amhara region (excluding South Wollo and North Shewa), Benishangul Gumuz, Wellega, Gambela (Nuer), Tigray, and Afar (Zone 2) (S2 Fig).

The proportion of children without a vaccination card showed a consistently strong and positive association across all areas (β = 0.509 to 0.648), indicating a key determinant of incomplete vaccination (S3 Fig). Similarly, home delivery was positively associated with incomplete vaccination in all areas (β: 0.171 to 0.238), with stronger effects observed in central regions (S4 Fig). Household size with more than five members also showed consistent significant positive effects in all areas (β = 0.091 to 0.099) (S5 Fig). In contrast, the proportion of children whose parents are not the head of household showed a negative association across all areas (β = −0.109 to −0.091) (S6 Fig).

## Discussion

This Study analyses the spatial distribution of newly introduced vaccine incomplete uptake and its associated factors among 12–35 month-aged children in Ethiopia. Overall, the prevalence of incomplete PCV and RVV uptake is 48.83%. This study shows that there is significant spatial clustering of incomplete PCV and RVV uptake. The hotspot analysis and interpolated prevalence also indicate the existence of vacillating incomplete intake of PCV and RVV in the Afar region, Somali region, and the North East part of SNNPR. Spatial scan analysis also indicates the existence of significant 3-cluster areas with a total of 98 enumeration areas within them. Previous studies also support the spatial variability of childhood vaccinations. A study performed on the spatial distribution of low BCG coverage shows significant clustering in the Afar region, Somali region, and the North East part of SNNPR [47,73,84]. A previous study performed on the spatial distribution of not having all age-appropriate vaccination coverage reveals that such hot spot clustering is found in afar regions, the eastern part of the country, and the northeastern SNNPR region [85]. This spatial pattern often points to the disadvantaged status of the pastoralist and agrarian regions in the eastern and southern parts of the country. Other studies support this study finding, as these regions grapple with chronic poverty, limited educational and economic opportunities, and persistently weak health system infrastructure, all of which can constrain the uptake of newly introduced PCV and RVV vaccines [12,19,86].

Our study shows a highly significant association between vaccination card availability and incomplete vaccination for newly introduced vaccines in all of Ethiopia. Vaccination cards can be utilized by caregivers as a tool for following their child’s immunization status and schedule [87]. Previous studies performed in Africa and China also showed such an impact of vaccination card availability on childhood vaccination status [30,33,37,38,40]. Large household size is also another significant predictor for incomplete newly introduced vaccine uptake. This might be because there is a logistical and resource shortfall in families with larger household sizes. Previous studies also support the negative influence of higher family size on full vaccination status [25,71,88–91]. Some studies also show no implication of household member size in vaccination status [92].

Our Study shows the existence of a significant negative association between parents not being the head of the household and not getting the full dosage of newly introduced vaccines. This means that if the parents of the children are not the heads of the house, the children will have a better chance of getting the full dose. This might be due to reduced authority, which turns the attention of the caregiver to health and vice versa [93]. This finding contradicts a study performed in Gambia on unvaccination for full vaccines [47]. The difference might be due to changes in population context, design, and methodological differences or due to different health systems and policy environments. Being home-delivered children is also another significant spatial determinant for incomplete newly introduced vaccine uptake. Its effect shows variability across Ethiopia, with its highest effect encountered around the capital city and surrounding areas. This result aligns with the previous study [25,40,47,61,64,89].

Our study also shows that not having PNC in the first two months of the life of children is another significant spatial factor that determines incomplete vaccination uptake for newly introduced vaccines. Its spatial distribution is explained by being markedly significant in the northern and northwest areas of the country only, encompassing the Tigray region, Afar (Zones 2), Amhara (Gondar, North Wollo, Wag Himra, Agew, and West Gojam), and Benishangul-Gumuz. Multilevel Study and Study performed in northwest Ethiopia also supported this finding [37,72,87,94,95]. A previous EDHS 2011 analytical study reveals such an association, whereas a multilevel analysis of only PCV vaccine uptake using 2016 EDHS shows non-significance of PNC [37,64]. Also, another systematic review study shows that non-significance of PNC in vaccination status, this might be due to the inclusion of studies performed in the central and southern part, where it is not significant, as our Study shows [89]. Being from mothers who have early maternal age at first birth have spatial varying effects on uptake of newly introduced vaccines. In the west, north west and south west it doesn’t have an effect, but in the rest part it has varying effects. Its effect gets pronounced as we go from west to east. Previous Study also concluded that older mothers tend to vaccinate their children which might be explained by maternal maturity [87].

## Strength and limitation of the Study

The analysis is based on utilizing nationally representative data but perhaps there is a limitation that is traced back to EDHS limitation like recall bias and social desirability bias. The Study used a Bayesian interpolation technique for predicting the prevalence all over the country, which reduces false positivity and false negativity. The Study included application of spatial regression analysis to health-related factors and outcome, that can provide valid estimates while adjusting for the existing spatial autocorrelation. Perhaps also the existence of mitigation of actual GPS coordinates for the purpose of anonymity might influence the hot and cold spot local estimation. To bypass such limitations, the Study focused on high-level interpretation. We also acknowledge that the Jarque–Bera test, used for normality assessment, has lower power for detecting platykurtic distributions like ours compared to alternative tests.

## Conclusion

This study demonstrated that incomplete uptake of newly introduced vaccines among children in Ethiopia is spatially clustered, with clear geographic inequalities. The findings highlight that gaps in maternal healthcare utilization and documentation practices play a central role in shaping vaccination coverage. To address these disparities, targeted and area-specific interventions are essential. Strengthening postnatal care utilization, improving access to and retention of vaccination cards, and promoting institutional delivery should be prioritized, particularly in identified hotspot regions. In addition, community-based awareness programs and strategies aimed at reducing early maternal age at first birth and managing household resource constraints could further enhance vaccine uptake. Overall, a geographically tailored public health approach that integrates maternal health services with immunization programs is critical to improving equitable vaccine coverage in Ethiopia.

## Supporting information

S1 Table Spatial scan analysis results of incomplete newly introduced childhood vaccination among children aged 12–35 months in Ethiopia, 2019. Map image is the intellectual property of Esri and is used herein under license. Copyright © 2026 Esri and its licensors. All rights reserved.
(DOCX)

S1 Fig The MGWR coefficients and spatially varying significant level of less than 18 maternal age at first birth on predicting incomplete newly introduced childhood vaccination among children aged 12–35 months in Ethiopia, 2019. Map image is the intellectual property of Esri and is used herein under license. Copyright © 2026 Esri and its licensors. All rights reserved.
(TIF)

S2 Fig The MGWR coefficients and spatially varying significant level of delayed or absent postnatal care on predicting incomplete newly introduced childhood vaccination among children aged 12–35 months in Ethiopia, 2019. Map image is the intellectual property of Esri and is used herein under license. Copyright © 2026 Esri and its licensors. All rights reserved.
(TIF)

S3 Fig The MGWR coefficients and spatially varying significant level of availability of a child’s vaccination card predicting incomplete uptake of newly introduced vaccines among children aged 12–35 months in Ethiopia, 2019. Map image is the intellectual property of Esri and is used herein under license. Copyright © 2026 Esri and its licensors. All rights reserved.
(TIF)

S4 Fig The MGWR coefficients and spatially varying significant level of home delivery on predicting incomplete newly introduced childhood vaccination among children aged 12–35 months in Ethiopia, 2019. Map image is the intellectual property of Esri and is used herein under license. Copyright © 2026 Esri and its licensors. All rights reserved.
(TIF)

S5 Fig The MGWR coefficients and spatially varying significant level of Household size predicting incomplete newly introduced childhood vaccination among children aged 12–35 months in Ethiopia, 2019. Map image is the intellectual property of Esri and is used herein under license. Copyright © 2026 Esri and its licensors. All rights reserved.
(TIF)

S6 Fig The MGWR coefficients and spatially varying significant level of parent not being head of the household predicting incomplete newly introduced childhood vaccination among children aged 12–35 months in Ethiopia, 2019. Map image is the intellectual property of Esri and is used herein under license. Copyright © 2026 Esri and its licensors. All rights reserved.
(TIF)

## Abbreviations

AIC: Akaike Information Criterion
AICc: Corrected Akaike Information Criterion
ANC: Antenatal Care
BIC: Bayesian Information Criterion
DHS: Demographic and Health Survey
EA(s): Enumeration Area(s)
EBK: Empirical Bayesian Kriging
EMDHS: Ethiopian Mini Demographic and Health Survey
GSS: Golden Section Search
GWBR: Geographically Weighted Beta Regression
IDW: Inverse Distance Weighting
IQR: Interquartile Range
KR: Kids Record
LLR: Log Likelihood Ratio
LM: Lagrange Multiplier
LR: Likelihood Ratio
MGWR: Multi-scale Geographically Weighted Regression
OLS: Ordinary Least Squares
PCV: Pneumococcal Conjugate Vaccine
PNC: Postnatal Care
RR: Relative Risk
RVV: Rotavirus Vaccine
SD: Standard Deviation
SEM: Spatial Error Model
SLM: Spatial Lag Model
VIF: Variance Inflation Factor
