# Supplementary material for: Spatial distribution of uptake of newly introduced vaccines and its associated factors among children aged 12–35 months in Ethiopia: Multi-scale Geographically Weighted Regression Analysis
Source: PLoS One. 2026 Jul 2;21(7):e0345899. doi: 10.1371/journal.pone.0345899 (PMC13327192; doi:10.1371/journal.pone.0345899)
Supplement: S1 Table — Map image is the intellectual property of Esri and is used herein under license. Copyright © 2026 Esri and its licensors. All rights reserved. (DOCX) [file pone.0345899.s001.docx]

S1 Table 1: Spatial scan analysis results of incomplete newly introduced childhood vaccination among children aged 12–35 months in Ethiopia, 2019

|  | **Cluster Type** | | |
| --- | --- | --- | --- |
|  | **Primary Cluster** | **Secondary Cluster** | **Tertiary Cluster** |
| **Significant Clusters**  **Detected** | 144, 141, 125, 143, 142, 114, 136, 138, 137, 111, 89, 113, 123, 110,183, 117, 134, 188, 186, 172, 181, 182, 115, 185, 103, 187, 145, 202, 184, 135, 197, 133, 116, 199, 178, 102, 190, 198, 104, 203, 131, 189, 191, 180, 205, 105, 175, 88, 173, 90, 106, 177, 192, 179, 204, 193, 101, 28, 122, 176, 41, 196, 140, 132, 250, 108, 248, 244, 129, 247, 249, 234, 233, 171, 243, 241, 252, 246, 237, 255, 245, 231, 235, 242, 127, 240, 239, 236, 232, 278, 238, 280, 254, 253, 107 | 95 | 99, 100 |
| **Coordinates / Radius** | (4.028421 N, 41.180723 E) / 601.08 km | (8.039877 N, 37.283375 E) / 0 km | (9.531226 N, 38.081685 E) / 67.38 km |
| **Population** | 988 | 21 | 40 |
| **Number of Cases** | 626 | 21 | 32 |
| **Relative risk** | 1.83 | 2.08 | 1.67 |
| **Percent cases in area** | 63.4 | 100.0 | 80.0 |
| **Log Likelihood Ratio** | 85.30 | 15.29 | 8.58 |
| **P-Value** | 0.001 | 0.001 | 0.023 |
